# Reproductive output of a non-zooxanthellate temperate coral is unaffected by temperature along an extended latitudinal gradient

**DOI:** 10.1371/journal.pone.0171051

**Published:** 2017-02-03

**Authors:** Valentina Airi, Selena Prantoni, Marco Calegari, Veronica Lisini Baldi, Francesca Gizzi, Chiara Marchini, Oren Levy, Giuseppe Falini, Zvy Dubinsky, Stefano Goffredo

**Affiliations:** 1 Marine Science Group, Department of Biological, Geological and Environmental Sciences, University of Bologna, Bologna, Italy, European Union; 2 The Mina and Everard Goodman Faculty of Life Sciences, Bar-Ilan University, Ramat-Gan, Israel; 3 Department of Chemistry “Giacomo Ciamician”, University of Bologna, Bologna, Italy, European Union; US Geological Survey, UNITED STATES

## Abstract

Global environmental change, in marine ecosystems, is associated with concurrent shifts in water temperature, circulation, stratification, and nutrient input, with potentially wide-ranging biological effects. Variations in seawater temperature might alter physiological functioning, reproductive efficiency, and demographic traits of marine organisms, leading to shifts in population size and abundance. Differences in temperature tolerances between organisms can identify individual and ecological characteristics, which make corals able to persist and adapt in a climate change context. Here we investigated the possible effect of temperature on the reproductive output of the solitary non-zooxanthellate temperate coral *Leptopsammia pruvoti*, along an 8° latitudinal gradient. Samples have been collected in six populations along the gradient and each polyp was examined using histological and cyto-histometric analyses. We coupled our results with previous studies on the growth, demography, and calcification of *L*. *pruvoti* along the same temperature gradient, and compared them with those of another sympatric zooxanthellate coral *Balanophyllia europaea* to understand which trophic strategy makes the coral more tolerant to increasing temperature. The non-zooxanthellate species seemed to be quite tolerant to temperature increases, probably due to the lack of the symbiosis with zooxanthellae. To our knowledge, this is the first field investigation of the relationship between reproductive output and temperature increase of a temperate asymbiotic coral, providing novel insights into the poorly studied non-zooxanthellate scleractinians.

## Introduction

Anthropogenic-derived climate change is the main source of environmental perturbation on a global scale, with an accelerated rate of increasing temperature that exceeds many pessimistic projections [1]. Combined with rising sea levels and shifting weather patterns, warming will have significant impacts on global biodiversity and ecological functioning [1, 2]. Given the substantial impact of climate change on ecological communities [3], studying how environmental parameters affect population persistence, community structure and the sustainable delivery of ecosystem services is a major challenge for conservation biology and ecosystem management [4].

The Mediterranean is one of the regions most affected by the ongoing global warming [5, 6] where extreme climatic events are becoming more frequent, faunas are shifting, and invasive species are spreading [6]. Given these facts, the Mediterranean Sea can act as a giant mesocosm of the world’s oceans with many sources of disturbances interacting synergistically [5] and represents an excellent natural laboratory for exploring the responses of temperate marine biota in relation to climate change [5, 6].

Temperature is a key environmental factor in marine ecosystems, affecting organisms at all organizational levels by controlling their physiological and ecological processes [7, 8]. Increasing seawater temperature is likely to affect population biology of coral species by reducing reproductive efficiency [9]. Reproductive output can be defined as the average product of gamete abundance representing the reproductive effort of an individual [10], which is strongly related to intrinsic population growth rate and demography [11]. Without successful reproduction, coral populations are unable to replenish lost individuals, leading to a possible regional extinction [12].

Despite the wealth of information on sexual reproduction in scleractinians, few studies have been performed on the Mediterranean corals [13]. Moreover, most of the researches about the effects of environmental parameters on sexual reproduction refer to tropical symbiotic corals (e.g. [14, 15]), which seem particularly sensitive to elevated temperatures [16, 17]. Conversely, the influences of increasing temperature on non-zooxanthellate corals have been scarcely investigated.

This study focused on the solitary non-zooxanthellate dendrophylliid *Leptopsammia pruvoti*, Lacaze-Duthiers, 1897 (S1 Fig), a gonochoric and brooding coral [18, 19]. Our specific aim was to quantify the reproductive output of *L*. *pruvoti* along a latitudinal gradient, in order to investigate the effects of temperature on the non-zooxanthellate coral reproduction. Latitude is the main factor influencing solar radiation and consequently temperature variations [20], which have already shown correlations with biological parameters of *L*. *pruvoti* [21] and other temperate dendrophylliids [21–27]. Previous studies on this species report no influence of temperature on population abundance, skeletal architecture features (such as corallite length, width, height, and bulk density [21, 23], population dynamics [25] and calcification [26]. However, the calcium carbonate density of its skeleton seems to increase with higher temperatures [21].

An analogous study was conducted on the endemic Mediterranean zooxanthellate solitary coral *Balanophyllia europaea* (Risso, 1826), collected at the same sites, during the same time interval and using the same methods of the present study. *B*. *europaea* experienced lower ecological performance in warmer populations that show a loss of oocytes during gametogenesis, probably due to their reabsorption in order to allocate the energy towards other vital functions [27]. Moreover, the warmer populations display a lower growth coefficient [24] and a lower population density [22, 23], as a possible negative effect of high temperature on the photosynthetic efficiency of the zooxanthellae [28].

In light of these previous findings, we expect to find a controversial response of *L*. *pruvoti*: does temperature not affect the reproductive output, according to analyzed growth and population dynamics, or does it negatively affect gametogenesis, as shown in the sympatric zooxanthellate coral *B*. *europaea*?

## Materials and methods

### Ethics statement

According to the European normative (2010/63/EU of 8 August 2010) on the protection of animals used for scientific purposes, there is no active conservation measure for the Mediterranean coral *L*. *pruvoti*. The species is not protected in Italy, nor it is subject to any regulations. Hence, no permit was needed to collect samples. For this study, sampling was limited strictly to the number necessary and performed where the species is characterized by a high population density to minimize the impact of removing individuals and preserve both the demographic and genetic structure of the natural populations.

### Sample collection

**Fig 1:**
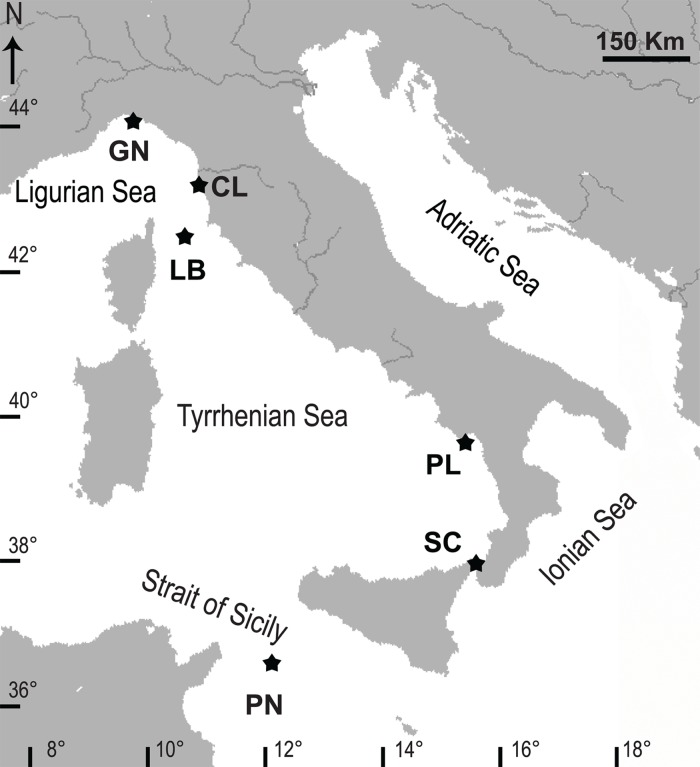
Map of the Italian coastline indicating the sites where corals have been collected. Abbreviations and coordinates of the sites in decreasing order of latitude: GN, Genova, 44°20’N, 9°08’E; CL, Calafuria, 43°27’N, 10°21’E; LB, Elba Isle, 42°45’N, 10°24’E; PL, Palinuro, 40°02’N, 15°16’E; SC, Scilla, 38°01’N, 15°38’E; PN, Pantelleria Isle, 36°45’N, 11°57’E.

Specimens of *L*. *pruvoti* were collected from six sites along a latitudinal gradient, from 44°20'N to 36°45'N (Fig 1). Reproductive data of Calafuria came from a previous study [19]. Approximately 15 polyps were collected each month between June 2010 and November 2012 from five populations (Genova, Elba Isle, Palinuro, Scilla and Pantelleria) and between July 2001 and September 2002 from the Calafuria population. For each sampling, individuals with an oral disc larger than 5 mm (sexually matures [19]) have been chosen.

Polyps were collected between 15–17 m depth by SCUBA diving, where populations were characterized by high population density [23]. Additional data on the species, e.g., biometry, population density, growth (calcification rate, linear extension rate, and skeletal density), population dynamics, and genetics have been examined in previous studies [21, 23, 25, 26, 29, 30].

### Biometric, histological and cytometric analyses

Biometric analyses were performed by measuring length (L, maximum axis of the oral disc), width (W, minimum axis of the oral disc), and height (h, oral–aboral axis) of each sampled polyp. Polyp body volume (V), calculated using the formula V = (L/2) * (W/2) * h * π, was used to estimate reproductive parameters (oocyte and spermary abundance, and gonadal index) as described in the next section. Polyps have been processed following the histological protocols as described in Goffredo et al. [18, 19].

Cytometric analyses were carried out with an optical microscope using the image analyzer NIKON NIS-Elements D 3.2. The maximum and minimum diameters of the oocytes in nucleated sections and spermaries were measured. Spermaries were classified into five developmental stages and oocytes were classified as immature or mature, according to earlier studies on gametogenesis of this species [18].

### Reproductive parameters

Reproductive output was defined through three reproductive parameters: a) *oocyte* and *spermary abundance*, both defined as the number of reproductive elements per body volume unit (100 mm^3^); b) *gonadal index*, defined as the percentage of body volume occupied by reproductive elements [19]; and c) *reproductive element size*, defined as the mean of the maximum and minimum diameter of spermaries, and oocytes in nucleated section [19].

The reproductive year was characterized by two gamete activity periods [19]. The *gamete recruitment period* [31], corresponding to the generation of primary gametocytes, occurs in this species between June and September and is characterized by i) a stock of smaller oocytes and the recruitment of new ones; ii) the beginning of spermary development [19]. The *gamete maturity period* [32] takes place between December and March and is characterized by the presence of larger oocytes and an advanced spermary maturation stage [19].

### Environmental parameters

Depth temperature (DT; °C) was measured by digital thermometers (I-Button DS1921H, Maxim Integrated Products, Dallas Semiconductors, USA) placed close to the sampling site in each population. Sensors recorded DT every 3 hours during the entire experimental period. Thermometers were replaced every 3 months to avoid problems of encrustation and overgrowth by marine organisms.

Sea surface temperature (SST; °C) data for each site were obtained from the National Mareographic Network of the Institute for the Environmental Protection and Research (ISPRA, [33]). Mean annual SST values were computed from hourly measurements.

A linear regression was obtained between DT and SST data to estimate historical at-depth temperatures. For Calafuria population the regression has been obtained using SST and DT data from the same period of sample collection.

Solar Radiation (SR; W/m^2^) was taken from the Satellite Application Facility on Climate Monitoring (CM-SAF/EUMETSAT, [34]).

In this study, we considered an average DT and SR of 3 years (36 months including the 18 months of sampling interval).

### Statistical analyses

Levene’s test was used for testing homogeneity of variance and one-sample Kolmogorov-Smirnov test was used for testing normal distribution of data. The two-sample Kolmogorov-Smirnov test was used to compare the size-frequency distribution of reproductive elements between the two periods. Student’s *t* test was used to compare the mean oocyte and spermary size of populations between periods. One-way analysis of variance (ANOVA) or the non-parametric Kruskal-Wallis test was used to compare environmental and reproductive parameters among sites. In order to examine the effects of each environmental parameter (DT and SR) and their interaction (DT*SR) on reproductive output, a General Linear Model (GLM) was used. Data non-normally distributed have been analyzed by the Generalized Linear Models (GLM) with a log link function. In these models, reproductive parameters were the dependent variables, and environmental parameters were selected as covariates. Statistical analysis was performed using SPSS 22 (IBM SPSS Statistical, Chicago, USA).

## Results

Mean annual DT (°C) and mean annual SR (W/m^2^) were significantly different among sites (monthly DT, χ^2^ = 13.16, df = 5 and p-value < 0.05 in Kruskal-Wallis test; monthly SR, F = 84.36, df = 5 and p-value < 0.001 in ANOVA test; Table 1).

**Table 1:** Mean annual depth temperature (DT; °C) and solar radiation (SR; W/m^2^) from the sampled populations.

Population	Code	Mean DT	SE	Range DT	Mean SR	SE	Range SR
Calafuria	CL	17.30	0.48	13.27–21.93	178.8	16.0	48.0–340.0
Elba	LB	17.57	0.64	11.82–24.27	184.9	15.4	45.9–337.9
Genova	GN	17.69	0.56	12.42–22.58	156.9	13.4	36.0–290.5
Scilla	SC	18.14	0.48	14.05–22.89	205.5	14.8	72.5–338.6
Palinuro	PL	18.74	0.56	14.25–24.45	194.6	15.5	63.8–331.9
Pantelleria	PN	18.99	0.58	14.14–25.39	218.2	14.2	91.2–351.1

The sites are arranged in order of increasing DT. SE, standard error; n for DT and SR = 36.

**Fig 2:**
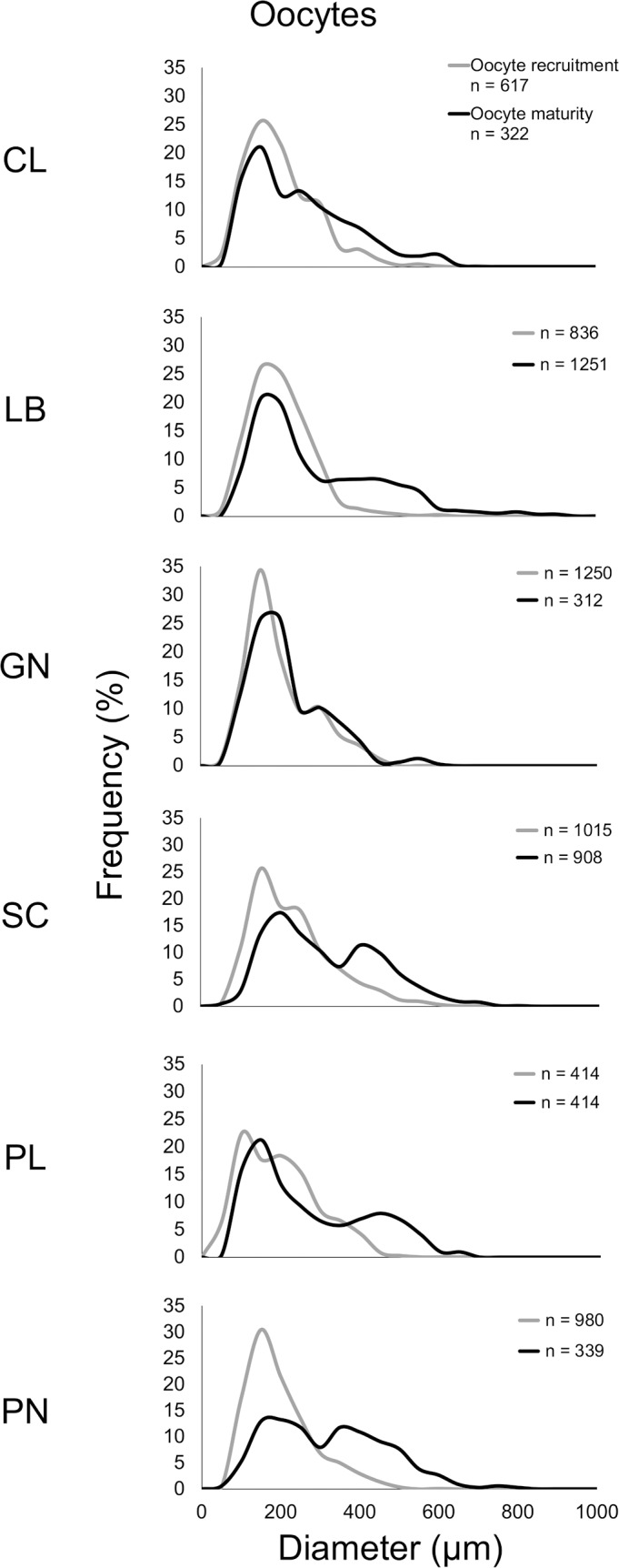
Oocyte size-frequency distribution. Distribution of oocyte size during the gamete recruitment period (grey line) and gamete maturity period (black line). Site codes in increasing order of DT (°C): CL, Calafuria; LB, Elba Isle; GN, Genova; SC, Scilla; PL, Palinuro; PN, Pantelleria Isle.

All populations showed gonochoric polyps in both reproductive periods. Size/frequency distribution during the gamete recruitment period was significantly different from that of the gamete maturity period in all populations (n = 6; Kolmogorov-Smirnov, p < 0.001, Genova (GN) population p < 0.05; Fig 2). Within the gamete recruitment period, most oocytes were smaller than 400 μm in all populations. In the following season (December-March), two distinct oocyte stocks appeared in all populations, characterized by small (immature < 400 μm) and large (mature > 400 μm) cells respectively (Fig 2). The mean oocyte size during the gamete recruitment period was significantly lower than the gamete maturity period in all populations (Table 2 for n value; Calafuria (CL), t = -6.53, df = 937 and p-value < 0.001; Elba (LB), t = -14.72, df = 2085 and p-value < 0.001; Genova (GN), t = -2.57, df = 920 and p-value < 0.05; Palinuro (PL), t = -8.82, df = 826 and p-value < 0.001; Scilla (SC), t = -15.51, df = 1921 and p-value < 0.001; Pantelleria (PN), t = -18.75, df = 1317 and p-value < 0.001 in Student’s *t*-test).

**Table 2:** Mean abundance, gonadal index, and diameter of oocytes in each population for both reproductive periods.

**Gamete recruitment period**					
**Population**	**n**	**Abundance (#/100 mm^3^) mean ± SE**	**Gonadal index (%) mean ± SE**	**n**_**o**_	**Diameter (μm) mean ± SE**
CL	19	580 ± 164	0.94 ± 0.18	617	177.0 ± 3.7
LB	7	673 ± 238	1.42 ± 0.48	836	175.8 ± 2.7
GN	6	1257 ± 298	2.82 ± 0.77	1250	180.8 ± 2.3
SC	5	1263 ± 517	3.67 ± 1.47	1015	204.2 ± 3.1
PL	6	692 ± 170	1.31 ± 0.41	414	170.1 ± 4.7
PN	5	1112 ± 311	2.19 ± 0.82	976	174.1 ± 2.7
**Gamete maturity period**					
**Population**	**n**	**Abundance (#/100 mm^3^) mean ± SE**	**Gonadal index (%) mean ± SE**	**n**_**o**_	**Diameter (μm) mean ± SE**
CL	11	630 ± 177	2.21 ± 0.49	322	224.7 ± 7.2
LB	9	602 ± 92	3.80 ± 0.84	1251	263.6 ± 4.5
GN	4	906 ± 304	1.89 ± 0.38	312	192.6 ± 5.5
SC	5	551 ± 114	4.72 ± 0.72	908	288.2 ± 4.5
PL	6	605 ± 139	3.13 ± 0.84	414	246.2 ± 7.2
PN	6	357 ± 114	3.16 ± 1.31	339	296.4 ± 7.8

The sites are arranged in order of increasing DT (°C). SE, standard error; n, polyp number for abundance and gonadal index; n_o_, oocyte number for diameter.

**Fig 3:**
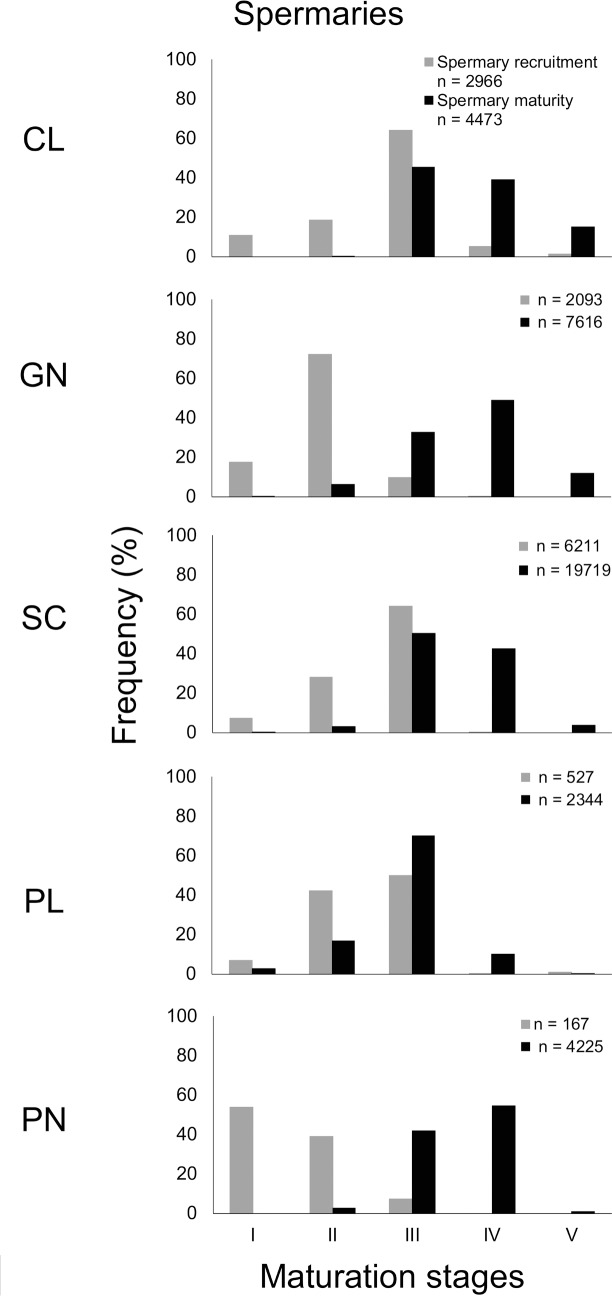
Spermary size-frequency distribution. Distribution of maturation stages during the gamete recruitment period (gray histogram bars) and gamete maturity period (black histogram bars). Site codes in increasing order of DT (°C): CL, Calafuria; LB, Elba Isle; GN, Genova; SC, Scilla; PL, Palinuro; PN, Pantelleria Isle. The Elba population was excluded from this analysis since male polyps were not found during the reproductive periods considered in this study.

Spermary maturation occurred progressively during the two periods. From the gamete recruitment period, each population was characterized, by small spermaries, mainly belonging to the earliest maturation stages (stages I, II and III). During the gamete maturity period, all populations were characterized by more advanced maturation stages (stages IV and V; Fig 3). The distribution of spermary maturation stages in the gamete recruitment period was significantly different from that of the gamete maturity period in all populations (Kolmogorov-Smirnov, p < 0.001; Fig 3). The mean spermary size during the gamete recruitment period was significantly lower than the gamete maturity period in all populations (Table 3 for n value; CL, t = -46.80, df = 7437 and p-value < 0.001; GN, t = -64.31, df = 9707 and p-value < 0.001; PL, t = -23.00, df = 2869 and p-value < 0.001; SC, t = -66.65, df = 25928 and p-value < 0.001; PN, t = -22.60, df = 4390 and p-value < 0.001 in Student’s *t*-test). The Elba population was excluded from this analysis since male polyps were not found during either reproductive periods considered in this study.

**Table 3:** Mean abundance, gonadal index, and diameter of spermaries in each population for both reproductive periods.

**Gamete recruitment period**					
**Population**	**n**	**Abundance (#/100 mm^3^) mean ± SE**	**Gonadal index (%) mean ± SE**	**n**_**s**_	**Diameter (μm) mean ± SE**
CL	27	3903 ± 765	0.60 ± 0.13	3019	68.0 ± 0.5
GN	6	6635 ± 1432	0.43 ± 0.09	2093	48.0 ± 0.4
SC	6	8270 ± 2692	1.00 ± 0.41	6211	68.6 ± 0.3
PL	6	3051 ± 1950	0.20 ± 0.13	527	53.9 ± 0.9
PN	4	1673 ± 704	0.05 ± 0.02	167	31.9 ± 0.9
**Gamete maturity period**					
**Population**	**n**	**Abundance (#/100 mm^3^) mean ± SE**	**Gonadal index (%) mean ± SE**	**n**_**s**_	**Diameter (μm) mean ± SE**
CL	13	12563 ± 2711	7.63 ± 1.46	4473	121.9 ± 0.9
GN	5	12686 ± 4254	8.78 ± 2.41	7616	126.7 ± 0.6
SC	6	18858 ± 3564	12.69 ± 2.53	19719	123.2 ± 0.4
PL	5	8990 ± 2076	2.64 ± 0.75	2344	87.2 ± 0.8
PN	5	10241 ± 2583	3.80 ± 1.12	4225	99.2 ± 0.6

The sites are arranged in order of increasing DT. SE, standard error; n, polyps number for abundance and gonadal index; n_s_, spermary number for diameter.

**Fig 4:**
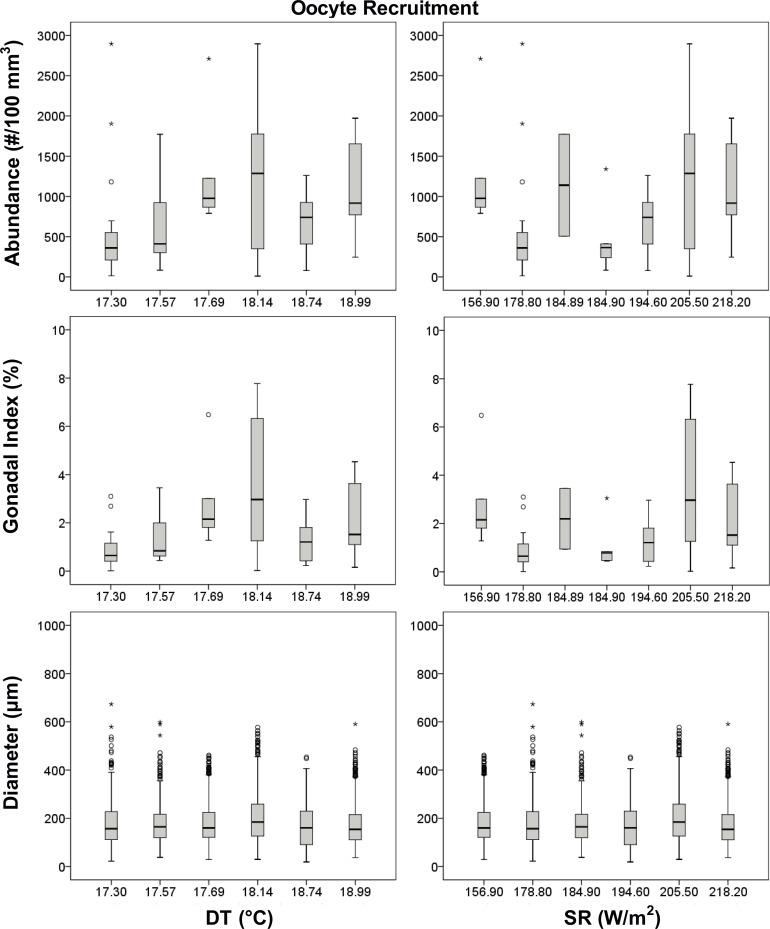
Oocytes boxplot in the gamete recruitment period. Boxplot between reproductive and environmental parameters during the gamete recruitment period. Median (solid horizontal line), first and third quartiles (box outline), minimum and maximum values (whiskers) and outliers (circles). See Table 2 for n values.

**Fig 5:**
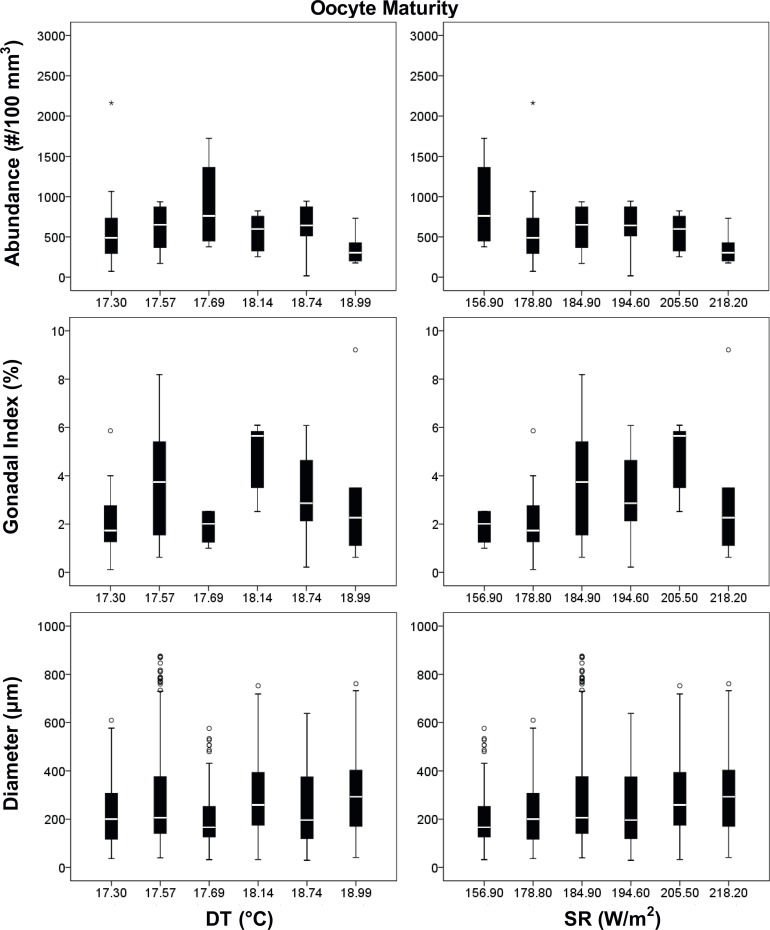
Oocytes boxplot in the gamete maturity period. Boxplot between reproductive and environmental parameters during the gamete maturity period. Median (solid horizontal line), first and third quartiles (box outline), minimum and maximum values (whiskers), and outliers (circles). See Table 2 for n values.

Oocyte diameter was significantly different among populations, during both periods (Kruskal–Wallis test, p < 0.001; Tables 2 and 4), resulting positively related to both environmental parameters (DT and SR) and negatively related to the interaction of both (DT*SR) only in the gamete recruitment period (Table 4, Figs 4 and 5).

**Table 4:** Oocytes analyses.

**Gamete recruitment period**				
		**DT (°C)**	**SR (W/m^2^)**	DT*SR
	**ANOVA–F**	**GLM—F (**β**)**	**GLM—F (**β**)**	**GLM—F (**β**)**
	**K-W—χ**^**2**^	**GLM—Wald χ**^**2**^ **(**β**)**	**GLM—Wald χ**^**2**^ **(**β**)**	**GLM—Wald χ**^**2**^ **(**β**)**
**Abundance (#/100 mm**^**3**^**)**	ns	-	-	-
**Gonadal index (%)**	ns	-	-	-
**Diameter (μm)**	(69.73)***	(0.67)***	(0.06)***	(-0.00)***
**Gamete maturity period**				
		**DT (°C)**	**SR (W/m^2^)**	DT*SR
	**ANOVA—F**	**GLM—F (**β**)**	**GLM—F (**β**)**	**GLM—F (**β**)**
	**K-W—χ**^**2**^	**GLM—Wald χ**^**2**^ **(**β**)**	**GLM—Wald χ**^**2**^ **(**β**)**	**GLM—Wald χ**^**2**^ **(**β**)**
**Abundance (#/100 mm**^**3**^**)**	ns	-	-	-
**Gonadal index (%)**	ns	-	-	-
**Diameter (μm)**	(171.28)***	ns	ns	ns

ANOVA/Kruskal-Wallis test and GLM analyses between reproductive and environmental parameters in the sampled populations in both periods. In brackets: F for ANOVA, χ^2^ for Kruskal-Wallis and β (regression coefficient) for GLM analyses.

* p < 0.05

** p < 0.01

*** p < 0.001

ns, not significant.

**Fig 6:**
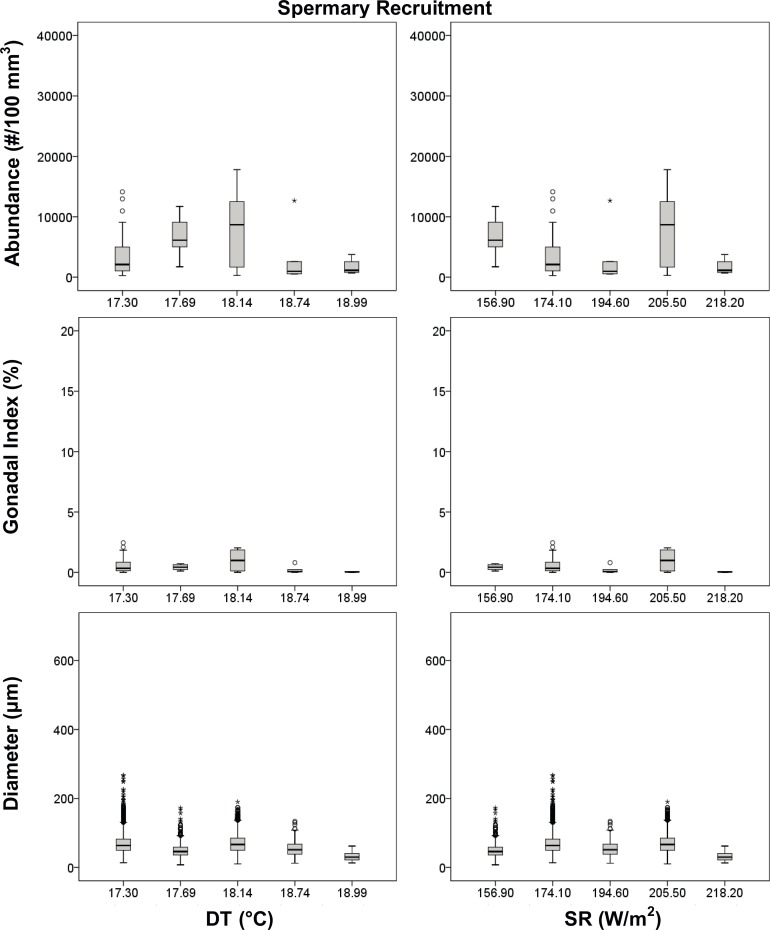
Spermaries boxplot in the gamete recruitment period. Boxplot between reproductive and environmental parameters during the gamete recruitment period. Median (solid horizontal line), first and third quartiles (box outline), minimum and maximum values (whiskers), and outliers (circles). See Table 3 for n values.

**Fig 7:**
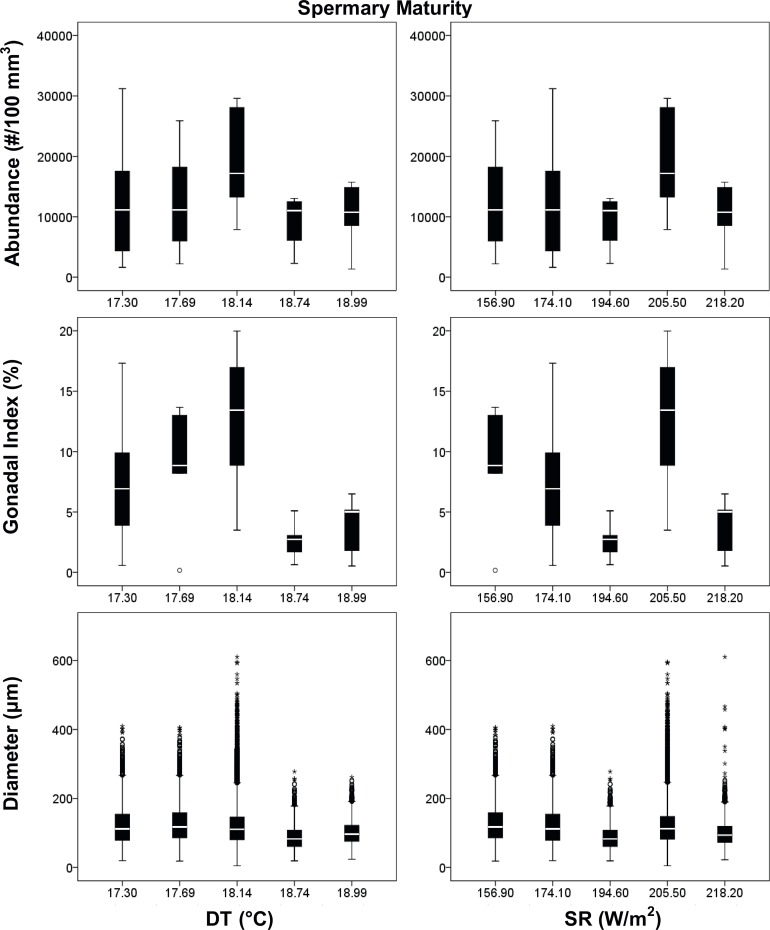
Spermaries boxplot in the gamete maturity period. Boxplot between reproductive and environmental parameters during the gamete maturity period. Median (solid horizontal line), first and third quartiles (box outline), minimum and maximum values (whiskers), and outliers (circles). See Table 3 for n values.

Spermary size was significantly different along the latitudinal gradient, during both periods (Kruskal–Wallis test, p < 0.001, Table 5). Spermary size increased with increasing DT and SR during the gamete recruitment period, resulting negatively related to their interaction (DT*SR) during the same period (Table 5, Fig 6). Instead, during the gamete maturity period spermary size was positively related to SR and negatively related to the interaction DT*SR (Table 5, Fig 7). Spermary gonadal index was significantly different among populations only during the gamete maturity period (ANOVA test, p < 0.01, Table 5) but was not related to both environmental parameters as well as to their interaction (Table 5, Fig 7).

**Table 5:** Spermaries analyses.

**Gamete recruitment period**				
		**DT (°C)**	**SR (W/m^2^)**	DT*SR
	**ANOVA—F**	**GLM—F (**β**)**	**GLM—F (**β**)**	**GLM—F (**β**)**
	**K-W—χ**^**2**^	**GLM—Wald χ**^**2**^ **(**β**)**	**GLM—Wald χ**^**2**^ **(**β**)**	**GLM—Wald χ**^**2**^ **(**β**)**
**Abundance (#/100 mm**^**3**^**)**	ns	-	-	-
**Gonadal index (%)**	ns	-	-	-
**Diameter (μm)**	(1600.36)***	(2.92)***	(0.33)***	(-0.02)***
**Gamete maturity period**				
		**DT (°C)**	**SR (W/m^2^)**	DT*SR
	**ANOVA—F**	**GLM—F (**β**)**	**GLM—F (**β**)**	**GLM—F (**β**)**
	**K-W—χ**^**2**^	**GLM—Wald χ**^**2**^ **(**β**)**	**GLM—Wald χ**^**2**^ **(**β**)**	**GLM—Wald χ**^**2**^ **(**β**)**
**Abundance (#/100 mm**^**3**^**)**	ns	-	-	-
**Gonadal index (%)**	(3.82)*	ns	ns	ns
**Diameter (μm)**	(1691.48)***	ns	(0.03)***	(-0.00)***

ANOVA/Kruskal-Wallis test and GLM analyses between reproductive and environmental parameters in the sampled populations in both periods. In brackets: F for ANOVA, χ^2^ for Kruskal-Wallis and β (regression coefficient) for GLM analyses.

* p < 0.05

** p < 0.01

*** p < 0.001; ns, not significant.

**Fig 8:**
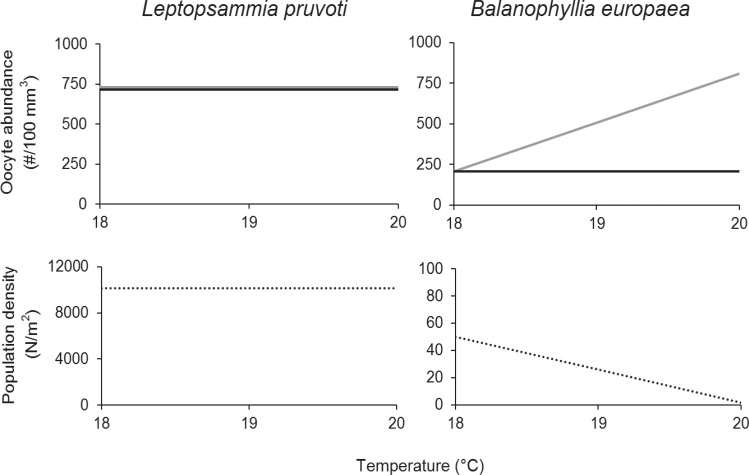
Schematic model of comparison between oocyte abundance and population density in *L*. *pruvoti* (non-zooxanthellate) and *B*. *europaea* (zooxanthellate) along the temperature gradient. Oocyte abundance during the gamete recruitment period (gray line) and the gamete maturity period (black line) in *L*. *pruvoti* (present study) and in *B*. *europaea* [27]. Population density (dashed line) in the same species [23].

While *L*. *pruvoti* was characterized by the same oocyte abundance along the temperature gradient in both periods (Figs 4 and 5 and Fig 8), in *B*. *europaea* the number of oocytes increased along the gradient during the gamete recruitment period, although it was constant during the gamete maturity period [27] (Fig 8).

In *L*. *pruvoti* oocyte abundance was not significantly different between the two reproductive periods (t = 1.68, df = 87 and p-value > 0.05 in Student’s *t*-test), while the warmer population of *B*. *europaea* showed an oocyte loss during the gametogenesis, experiencing a lower number of oocytes during the gamete maturity period, compared with the previous period (Fig 8). This loss increased along the gradient, showing a decrease of 59% at 19°C and 75% at 20°C, while the same oocyte abundance was reported at 18°C (Fig 8). Population density in *L*. *pruvoti* was constant along the gradient [23], whereas *B*. *europaea* showed lower population density in the warmer populations [23].

## Discussion

All populations along the gradient were characterized by a gonochoric sexuality with male and female polyps. However, the Elba Isle population showed only females, not allowing the analysis of spermatogenesis for this site.

Polyps of *L*. *pruvoti* seemed to show the same reproductive output in all the populations studied. In fact, oocyte and spermary abundance and female and male gonadal index were not affected by increasing DT and SR (Tables 4 and 5, Figs 4–7). Only the gamete diameter displayed a relation with both environmental parameters, probably due to the high amount of data (Tables 2 and 3). However, the medians shown in the box plots are almost overlapped (Figs 4–7), providing no biological interpretation.

These results are in agreement with previous findings concerning population density, growth, and population structure stability of this species, where these parameters were unaffected by SR and SST [21, 23, 25, 26]. *L*. *pruvoti* seemed to be quite tolerant to temperature variations compared to the zooxanthellate *B*. *europaea*, studied along the same gradient, whose warmer populations were less abundant, less stable (with loss of young individuals [21, 23, 24, 35] and were characterized by reduced efficiency in partitioning energy during gametogenesis [27].

The schematic model (Fig 8) summarizes the main findings on reproductive output and population density of these two species belonging to the same family and sharing a wide range of their distribution area. While in *L*. *pruvoti* oocyte abundance was the same during both periods, *B*. *europaea* invests more energy during the earliest stage of oocyte development, experiencing an oocyte loss during the gametogenesis that increased significantly along the gradient [27] (Fig 8). A possible explanation for this greater loss could be the inhibition of biological processes (such as growth [24], skeletal density [23], and calcification rate [35]) in the southern and warmer populations due to a less efficient symbiotic system [25], leading *B*. *europaea* to reabsorb a greater amount of oocytes in order to reallocate energetic resources towards other vital functions [27]. Nevertheless, both species showed the same reproductive output in the gamete maturity period in all populations (Fig 8), therefore, we expect to have the same population density along the gradient. This is the case for *L*. *pruvoti* [23] but not for *B*. *europaea*, characterized by lower population densities in the warmer populations, perhaps due to higher larval mortality [23]. *L*. *pruvoti* seems less sensitive to increasing temperature compared to *B*. *europaea*, which may be explained by the absence of symbionts in the former, and thus, the lack of inhibition of host physiological processes [23].

Environmental change can benefit some species with high rate of survival, growth and reproduction, allowing corals to adapt and persist in a changing world [7]. In many cases, however, a shift toward environmental conditions outside the normal range of variability is stressful, causing suboptimal physiological performance [36, 37]. For these individuals, more stressful conditions may lead to higher mortality, reduced growth, smaller size, and reduced reproduction [9]. For example, in zooxanthellate species thermal tolerance is primarily governed by the obligate relationship between the coral animal and its photosymbiotic partner [38], showing different efficiency under different environmental conditions, such as light and turbidity [39]. In turbid habitats, a heterotrophic metabolism is more advantageous [39] and under this condition non-zooxanthellate organisms could show greater adaptability. There is some evidence that enhanced stratification of coastal waters due to global warming is occurring in the Mediterranean Sea [40], causing turbidity of the water column, and consequent, limited light dispersal, negatively influencing the primary productivity of photosynthetic organisms [41]. This fact could lead non-zooxanthellate and zooxanthellate species to different degree of performance and adaptability to environmental change, suggesting that the heterotrophic *L*. *pruvoti* and the zooxanthellate *B*. *europaea* may have different ecological responses to the same temperature regime.

## Conclusion

*L*. *pruvoti* seems to be quite tolerant to the natural temperature range experienced in the field, since its organismal performance, such as population abundance, skeletal architecture and density, calcification rate [21, 23, 26], population dynamics [25], and reproductive output (present study), does not vary with temperature along the latitudinal gradient examined. However, the temperature threshold that this species will be able to tolerate is still unknown. Testing other environmental parameters not considered in this study, such as nutrients, zooplankton availability and turbidity, is crucial in order to verify the hypothesis that different responses between non-zooxanthellate (*L*. *pruvoti*) and zooxanthellate (*B*. *europaea*) species might depend on their different trophic systems. Further investigations are needed to better understand the environmental control on the ecology of these species, which may shed light on their potential resistance in the progressively warming Mediterranean Sea.

## Supporting information

S1 Fig *Leptopsammia pruvoti*. Living specimens of *Leptopsammia pruvoti* photographed at Scilla (South Italy, 38°01’N, 15°38’E). Photo by courtesy of Francesco Sesso.
(TIF)

S1 Dataset Raw data of oocyte reproductive parameters of *L*. *pruvoti* and *B*. *europaea*. Diameters of each oocyte and abundance and gonadal index of each polyp analysed in this study.
(XLS)

S2 Dataset Raw data of spermary reproductive parameters of *L*. *pruvoti*. Diameters of each spermary and abundance and gonadal index of each polyp analysed in this study.
(XLS)
