# Supplementary figures and images for: Reproductive output of a non-zooxanthellate temperate coral is unaffected by temperature along an extended latitudinal gradient
Source: PLoS One. 2017 Feb 3;12(2):e0171051. doi: 10.1371/journal.pone.0171051 (PMC5291506; doi:10.1371/journal.pone.0171051)

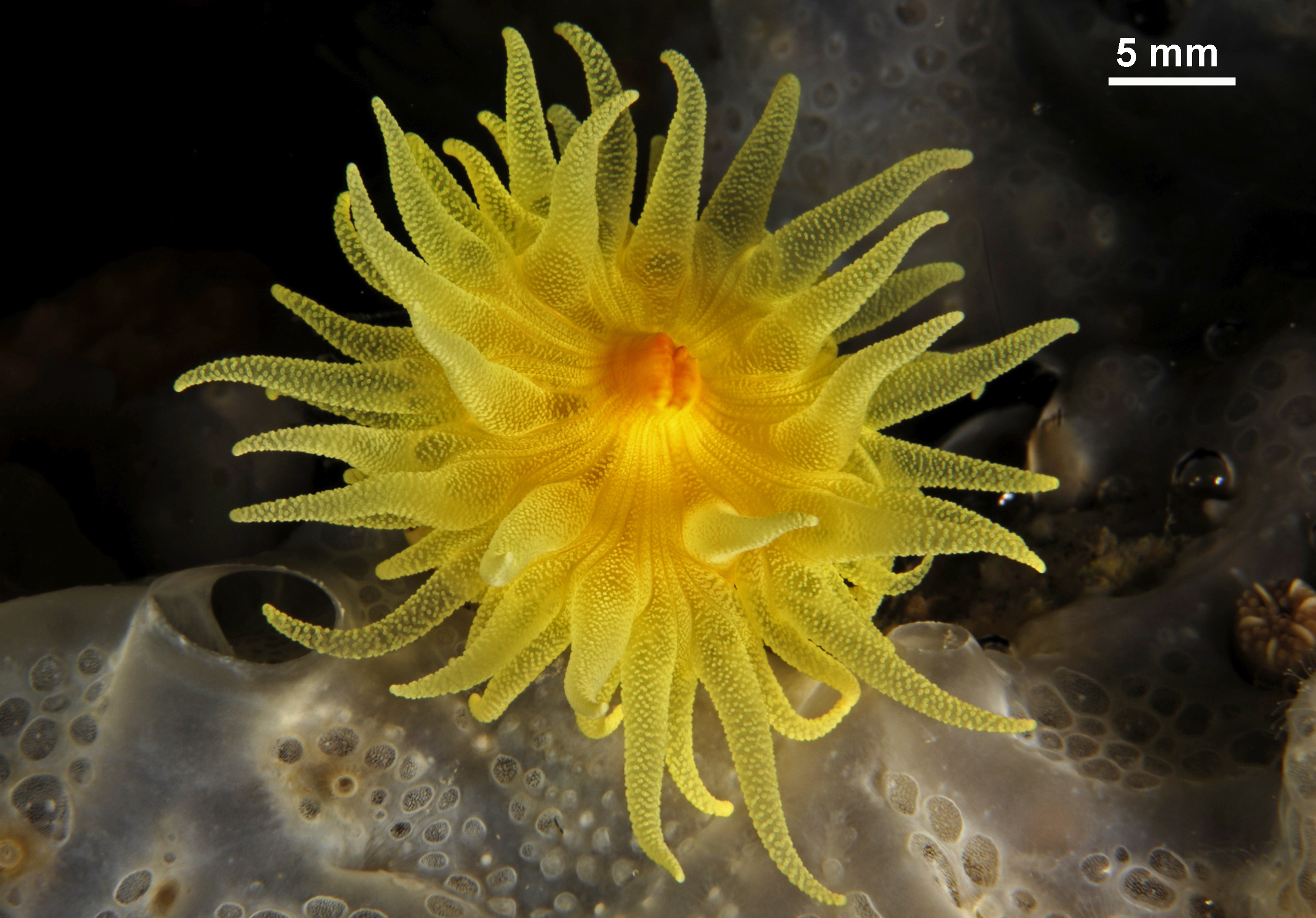

Supplement: S1 Fig — Living specimens of Leptopsammia pruvoti photographed at Scilla (South Italy, 38°01’N, 15°38’E). Photo by courtesy of Francesco Sesso. (TIF) [file pone.0171051.s001.tif]
